# Is Surgical Excision Mandatory for Sclerosing Adenosis Diagnosed on Core Needle Biopsy? Multimodal Imaging Features and Upgrade Outcomes in a Symptomatic Cohort

**DOI:** 10.3390/diagnostics16162570

**Published:** 2026-08-14

**Authors:** Abdulkadir Eren, Emrah Karatay, Ferhat Ozden

**Affiliations:** 1 Radiology, Istanbul Medipol University, Medipol Mega University Hospital, Istanbul 34214, Turkey; aeren@medipol.edu.tr; 2 Radiology, Sultan 2. Abdulhamid Han Training and Research Hospital, Istanbul 34668, Turkey; 3 Pathology, Istanbul Medipol University, Medipol Mega University Hospital, Istanbul 34214, Turkey; ferhat.ozden@medipol.edu.tr

**Keywords:** sclerosing adenosis, core needle biopsy, multimodality imaging, upgrade rate, high-risk breast lesion, B3 lesion, breast MRI

## Abstract

**Background**: Sclerosing adenosis (SA) is a benign, proliferative breast lesion that frequently mimics malignancy on multimodality imaging. The appropriate clinical management of SA diagnosed via core needle biopsy (CNB) remains a highly debated topic in breast oncology. This study aimed to evaluate the clinical, imaging, and histopathological characteristics of CNB-diagnosed SA-spectrum lesions and to determine the rate of, and factors associated with, pathological upgrade at the time of surgical excision in a symptomatic cohort. **Methods**: This retrospective, single-center study evaluated 34 symptomatic female patients who received a CNB diagnosis of SA. Patients were assessed using targeted ultrasound (US, *n* = 34), digital mammography (*n* = 19), and/or dynamic contrast-enhanced breast MRI (*n* = 17). Based on CNB findings, lesions were classified as isolated SA, complex/accompanied SA, or atypical SA. Patients subsequently underwent either definitive surgical excision or long-term imaging surveillance. Upgrades were defined as the presence of a high-risk B3 lesion not identified on CNB (Level 1) or overt malignancy, such as ductal carcinoma in situ or invasive carcinoma (Level 2), at final surgical pathology. **Results**: Twenty-one patients (61.8%) underwent surgical excision, while thirteen (38.2%) were managed with radiological follow-up (median surveillance 16 months, range 7–84). Within the surgical cohort, 9 of 21 patients (42.9%; 95% CI: 24.5–63.5%) demonstrated an upgrade: 6 (28.6%; 95% CI: 13.8–50.0%) to a B3-level lesion and 3 (14.3%; 95% CI: 5.0–34.6%) to malignancy. Notably, two of the three malignant upgrades originated from lesions initially classified as isolated SA without atypia on CNB. Due to the limited sample size, no single clinical or imaging variable (BI-RADS category, lesion size, or age) reached statistical significance as an independent predictor of upgrade (all *p* > 0.10). All patients managed with imaging surveillance remained radiologically stable. **Conclusions**: In stark contrast to the 1–2% upgrade rates traditionally reported in asymptomatic screening populations, symptomatic SA-spectrum lesions selected for surgical excision following CNB exhibited a substantially higher overall upgrade rate (42.9%) and malignant upgrade rate (14.3%) in our cohort, albeit with wide confidence intervals reflecting the modest surgical subgroup size. The occurrence of malignant upgrades from presumed isolated SA underscores the limitations of CNB sampling and highlights the necessity of multimodality imaging–pathology concordance. Because this estimate derives exclusively from patients already selected for surgery on the basis of clinical and imaging suspicion, it reflects the upgrade risk of this pre-selected, imaging-discordant subgroup and should not be extrapolated to the baseline risk of all CNB-diagnosed SA. Accordingly, these findings caution against extending non-operative management without further scrutiny to symptomatic SA cases showing a similar degree of imaging–pathology discordance: in such cohorts, surgical excision or large-volume vacuum-assisted excision may still merit consideration despite the absence of atypia on CNB, although this observation is drawn from a small, non-randomly selected surgical subgroup and should be interpreted with corresponding caution.

## 1. Introduction

Sclerosing adenosis (SA) is a complex, benign proliferative lesion originating from the terminal duct-lobular unit (TDLU) of the breast. Histopathologically, it is characterized by an enlarged, distorted lobular architecture featuring an increased number of acini that are compressed and distorted by dense, surrounding stromal fibrosis and collagen deposition. Despite its fundamentally benign cellular nature, SA is universally recognized in breast radiology as one of the “great mimickers” of carcinoma. The intense desmoplastic reaction it generates can present radiologically as an irregular, spiculated mass, a focal area of profound architectural distortion, or a cluster of pleomorphic microcalcifications. Consequently, SA frequently provokes a high index of clinical suspicion for invasive breast carcinoma or ductal carcinoma in situ (DCIS), leading to inevitable diagnostic interventions.

The optimal management of SA diagnosed via percutaneous core needle biopsy (CNB) represents a long-standing and polarizing point of debate within multidisciplinary tumor boards. Early observational series and conservative surgical guidelines suggested that isolated SA, when devoid of cellular atypia, carries an exceptionally low risk of upstaging to malignancy at the time of surgical excision [1,2]. These foundational studies supported the paradigm that short-term imaging follow-up is a safe and appropriate alternative to surgical intervention in cases where the imaging and pathology are deemed concordant. More contemporary series, primarily derived from large-scale, population-based screening programs, have reported similarly low malignant upgrade rates—typically hovering between 1 and 2%—when SA is the sole and dominant histological finding [3,4].

However, SA rarely manifests in absolute pathological isolation. It is frequently an integral component of a broader spectrum of complex proliferative diseases, routinely coexisting with other high-risk (B3) lesions such as radial scars (forming complex sclerosing lesions), intraductal papillomatosis, atypical ductal hyperplasia (ADH), and atypical lobular hyperplasia (ALH). For these concurrent entities, surgical excision is more aggressively indicated due to substantially higher established upgrade rates, which can exceed 20–30% when atypia is present [5,6,7]. This profound histological heterogeneity dictates that the solitary label of “sclerosing adenosis” on a standard pathology report may correspond to vastly different clinical risk profiles depending on adjacent, potentially unsampled microscopic findings.

Furthermore, the advent and widespread integration of advanced multimodality imaging—specifically Digital Breast Tomosynthesis (DBT), high-resolution automated breast ultrasound (ABUS), and dynamic contrast-enhanced magnetic resonance imaging (DCE-MRI)—have fundamentally altered the diagnostic landscape. MRI, for example, provides unparalleled sensitivity for breast cancer detection but often yields false-positive results in the presence of SA due to the lesion’s propensity for robust, early contrast enhancement and variable kinetic washout curves [8,9]. Radiologists and breast surgeons are thus frequently confronted with the dilemma of radiological–pathological discordance: a highly suspicious multimodality imaging phenotype (e.g., BI-RADS 4C or 5) juxtaposed with an ostensibly benign CNB diagnosis of isolated SA.

Crucially, the majority of the existing literature delineating SA upgrade rates is derived from asymptomatic, screening-based Western cohorts. Granular data describing the multimodality imaging–pathology correlation and surgical outcomes of CNB-diagnosed SA in symptomatic, non-screening populations remain markedly sparse. Symptomatic cohorts—such as those presenting to tertiary referral centers with palpable masses or focal breast pain—inherently carry a different pre-test probability for malignancy compared to their screening-detected counterparts [10,11]. Therefore, the primary objective of this study was to meticulously characterize the clinical and multimodality imaging features of CNB-diagnosed SA-spectrum lesions in a symptomatic cohort, and to determine the true upgrade rate at surgical excision to inform the ongoing debate of whether surgical excision can be safely omitted in this specific demographic. To the best of our knowledge, this study is among the first to report granular, multimodality imaging–pathology correlation and surgical upgrade outcomes specifically within a symptomatic, non-screening SA cohort, including detailed case-level analysis of the mechanisms by which malignancy evaded detection on initial core biopsy.

## 2. Materials and Methods

### 2.1. Study Design and Patient Population

This retrospective, single-center analytical study was conducted at the Department of Radiology, [Istanbul Medipol University Mega University Hospital ]. Institutional Review Board (IRB) approval was formally obtained from the [Istanbul Medipol University School of Medicine] Non-Interventional Clinical Research Ethics Committee prior to the commencement of data collection (Approval No: [E-11943279-202.4.02-7645, 24 June 2026]), and written informed consent has been obtained from the patients to publish this paper. A comprehensive, automated query of the institutional pathology and radiology informatics databases was executed to identify potential cases. To ensure a homogeneous and clinically relevant cohort, strict selection criteria were applied.

**Inclusion criteria were defined as follows:**

- Female patients who underwent percutaneous image-guided core needle biopsy (CNB) between 2020 and 2026.
- A primary histopathological diagnosis containing “sclerosing adenosis” on the CNB specimen, either isolated or accompanied by other benign or high-risk (B3) lesions.
- Availability of comprehensive pre-biopsy multimodality imaging (US, and/or MG, MRI).
- Documented follow-up consisting of definitive surgical excision or a minimum of 6 months of radiological surveillance.

**Exclusion criteria included the following:**

- Patients with a concurrent diagnosis of overt malignancy (DCIS or invasive carcinoma) definitively identified in the initial CNB specimen (as the primary objective was to evaluate occult upgrade risk).
- Patients with incomplete imaging records or lost to follow-up.

The initial data extraction based on these criteria yielded 35 instances. However, rigorous data cleaning and cross-referencing of protocol numbers identified one duplicate patient entry, which was systematically removed. Consequently, the final analytical cohort comprised 34 unique female patients. The overall patient flow is summarized as follows: 35 patients identified → 1 duplicate removed → 34 patients with CNB-diagnosed SA → 21 (61.8%) underwent surgical excision and 13 (38.2%) underwent radiological surveillance → within the surgical arm, 12 (57.1%) showed no upgrade, 6 (28.6%) showed a B3-level upgrade, and 3 (14.3%) showed a malignant upgrade → within the surveillance arm, all 13 (100%) remained radiologically stable through the available follow-up.

### 2.2. Multimodality Imaging Assessment

All patients underwent comprehensive pretreatment evaluation utilizing a tailored multimodality imaging approach based on their clinical presentation. The standard imaging protocol consisted of targeted, high-resolution breast ultrasound (US) performed on all patients using premium-tier diagnostic systems equipped with 12–18 MHz linear array transducers(GE Medical Systems, Glattbrugg, Switzerland). A subset of the cohort additionally underwent digital mammography (MG) and/or dynamic contrast-enhanced breast MRI, reflecting real-world clinical algorithms.

The Breast Imaging Reporting and Data System (BI-RADS) category (4A, 4B, 4C, or 5) assigned by the attending radiologist at the time of the initial biopsy was recorded. Imaging findings were systematically categorized: mammographic features were classified as architectural distortion, microcalcifications, solid mass, or focal asymmetry; sonographic features were classified as solid mass (often hypoechoic and irregular), posterior acoustic shadowing, architectural distortion, or cystic changes. The maximum lesional diameter was recorded based on the primary imaging modality utilized for biopsy guidance. For patients who underwent mammography, breast parenchymal density was classified according to the 5th edition of the ACR BI-RADS lexicon.

### 2.3. Percutaneous Biopsy and Pathological Classification

Image-guided percutaneous biopsy was performed under ultrasound guidance in 33 of 34 patients (97.1%) using a 14-gauge automated spring-loaded core biopsy device. In one patient (2.9%), MRI-guided vacuum-assisted biopsy (VAB) was utilized. The number of core tissue samples obtained per lesion was meticulously recorded.

Pathological specimens were evaluated by subspecialty-trained breast pathologists. When morphological ambiguity existed between the intense stromal sclerosis of SA and tubular or low-grade invasive carcinoma (the “pseudo-invasion” phenomenon), immunohistochemical (IHC) profiling—including markers such as p63, calponin, and smooth muscle myosin heavy chain (SMMHC)—was employed to confirm the integrity of the myoepithelial cell layer, ensuring an accurate diagnosis of SA. Based on the finalized CNB reports, lesions were stratified into three sub-cohorts: (1) isolated (“pure”) SA, demonstrating no accompanying high-risk architecture; (2) complex/accompanied SA, featuring concurrent radial scars, complex sclerosing lesions, complex fibroadenomas, or papillomatosis; and (3) atypical SA, demonstrating concurrent ADH or ALH.

### 2.4. Clinical Management and Outcome Definitions

Following multidisciplinary tumor board review, integrating the degree of imaging–pathology concordance, patients proceeded to either definitive surgical excision (wire-localized excisional biopsy or targeted segmentectomy) or conservative radiological follow-up.

The primary endpoint was the rate of pathological upgrade. Outcomes were defined hierarchically:

- **Level 0 (No Upgrade):** Final surgical pathology remained concordant with SA or other strictly benign entities.
- **Level 1 (B3-level Upgrade):** Identification of a high-risk lesion (e.g., ADH, ALH, or complex radial scar) at surgical excision that was definitively absent in the initial CNB specimen.
- **Level 2 (Malignant Upgrade):** Confirmation of DCIS or invasive breast carcinoma at final surgical pathology.

Patients managed non-operatively were deemed stable in the absence of morphological progression or suspicious changes on serial imaging during the surveillance interval.

### 2.5. Statistical Analysis

Descriptive statistics were generated for all baseline clinical and imaging variables. Continuous variables were expressed as means ± standard deviations or medians (with ranges), while categorical variables were presented as frequencies and percentages. To assess associations between categorical predictors (e.g., BI-RADS category, CNB classification) and the occurrence of any upgrade (Level 1 or Level 2) within the surgical cohort, Fisher’s exact test was utilized due to the small expected cell counts inherent to the sample size. Continuous variables (e.g., age, lesion size) were compared between the upgrade and no-upgrade cohorts using the non-parametric Mann–Whitney U test. A two-tailed *p*-value of <0.05 was considered indicative of statistical significance. Given the modest size of the surgical subgroup, 95% confidence intervals (CIs) for the overall, B3-level, and malignant upgrade proportions were calculated using the Wilson score method, which performs more reliably than the normal approximation for small samples and proportions near the extremes. Data compilation and statistical analyses were executed utilizing IBM SPSS Statistics for Windows (version 28.0; IBM Corp., Armonk, NY, USA) and Python (version 3.10; pandas and SciPy libraries, v1.13).

## 3. Results

### 3.1. Patient Demographics and Clinical Characteristics

The study population consisted of 34 female patients with a mean age of 36.6 ± 6.7 years (range: 28–54 years). Reflecting the tertiary care nature of the institution, all patients presented symptomatically: 18 patients (52.9%) presented with a clinically palpable mass, and 21 patients (61.8%) reported focal breast pain (with 5 patients experiencing both symptoms concurrently). No lesions within this cohort were detected incidentally via asymptomatic population screening. A positive family history of breast cancer was documented in 8 patients (23.5%). Multimodality imaging was extensively utilized; while all 34 patients underwent diagnostic ultrasound, 19 patients (55.9%) additionally received mammography, and 17 patients (50.0%) underwent breast MRI.

### 3.2. Multimodal Imaging Findings

The distribution of BI-RADS categories at presentation highlighted a broad spectrum of radiological suspicion. Over half of the lesions (*n* = 19, 55.9%) were classified as BI-RADS 4A. Lesions with higher indices of suspicion included BI-RADS 4B (*n* = 7, 20.6%) and BI-RADS 4C (*n* = 7, 20.6%). A single case (2.9%) presented with classic malignant morphology and was classified as BI-RADS 5. The mean maximal lesion diameter was 18.9 ± 11.5 mm (median: 15 mm, range: 4–58 mm).

Among the 19 patients evaluated with mammography, the background parenchymal density was predominantly dense (ACR Category C in 57.9%, and ACR Category D in 42.1%). On ultrasound, the predominant finding was a solid, irregular mass (31/34, 91.2%), often accompanied by posterior acoustic shadowing (8/34, 23.5%)—a hallmark of the intense fibrosis characteristic of SA. Mammographically, architectural distortion was the most frequent primary abnormality (9/19, 47.4%), followed by distinct mass formations (8/19, 42.1%) and suspicious microcalcifications (4/19, 21.1%).

### 3.3. CNB Classification and Surgical Management

Histopathological classification of the CNB specimens revealed that the majority of lesions (*n* = 22, 64.7%) were isolated (“pure”) SA. Complex or accompanied SA was identified in 10 patients (29.4%), with concurrent lesions including complex fibroadenomas (20.6%), papillomas (11.8%), and radial scars (5.9%). Atypical SA (concurrent ADH or ALH) was rare, present in only 2 patients (5.9%). The median number of core samples retrieved during the index biopsy was 7.5 (range: 3–20).

Subsequent to the CNB diagnosis, 21 patients (61.8%) underwent definitive surgical excision (8 excisional biopsies and 13 segmentectomies), while 13 patients (38.2%) were allocated to radiological surveillance.

### 3.4. Upgrade Outcomes and Pathological Concordance

**Figure 1:**
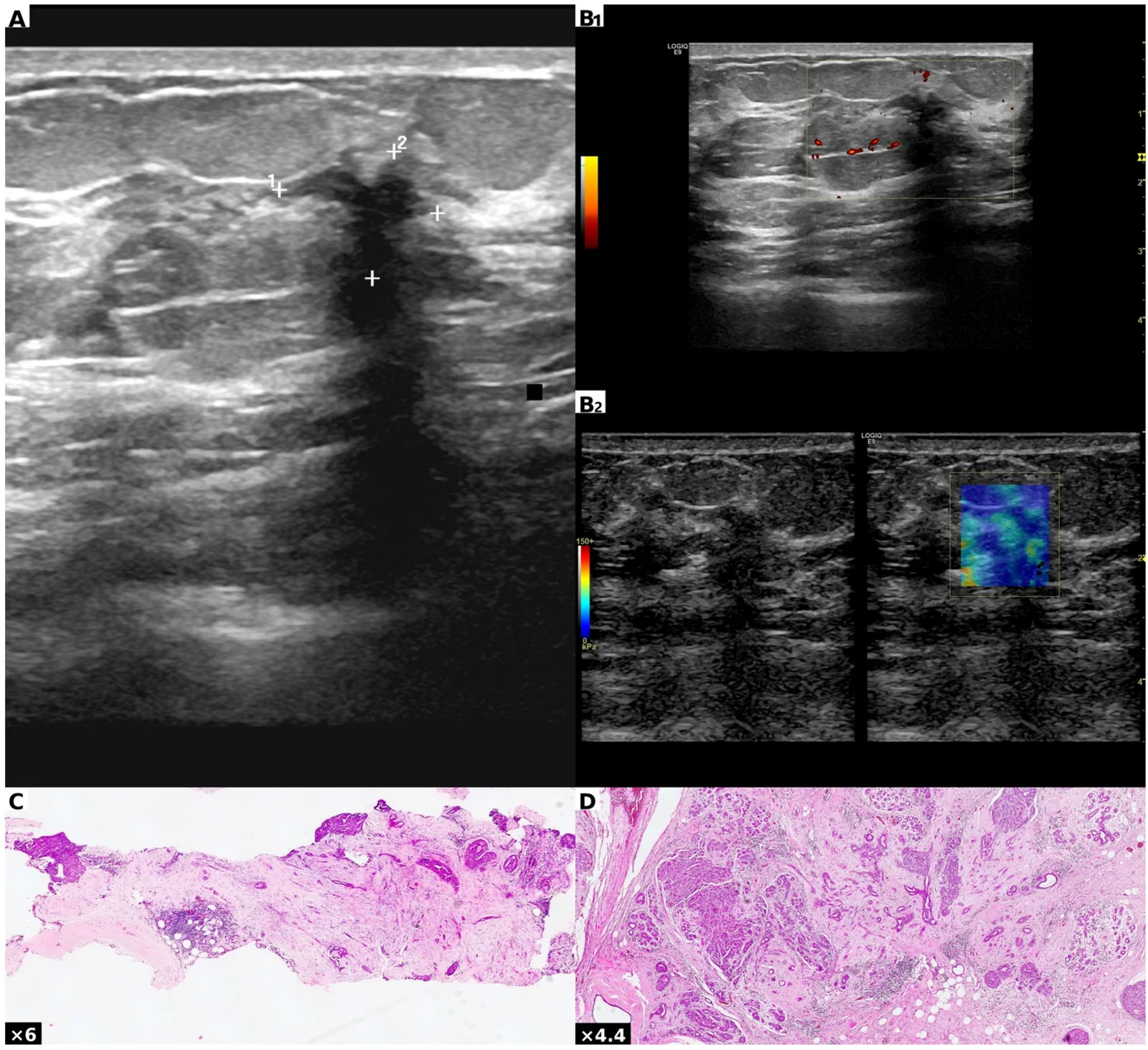
A 37-year-old female patient presenting with a 1-month history of a painful palpable mass, highlighting a radiologic–pathologic discordance that resulted in a high-risk B3 upgrade. (**A**) Targeted B-mode breast ultrasound reveals highly suspicious, irregular hypoechoic masses (largest measuring 11 × 9 mm) with distinct posterior acoustic shadowing (BI-RADS 4C). (**B_1_**) Color Doppler evaluation of the same lesion revealed no internal vascularity. (**B_2_**) Strain elastography mapping demonstrates predominantly soft tissue characteristics within the lesion, lacking the typical stiffness associated with malignancy. (**C**) Microscopic examination of the initial core needle biopsy specimen (H&E stain) yielded a benign diagnosis comprising isolated sclerosing adenosis (SA), ductal epithelial hyperplasia, and cystic changes. Immunohistochemical staining demonstrated an intact myoepithelial layer (p63-positive) and benign hyperplasia characteristics (CK5/6-positive, patchy ER positivity), lacking cellular atypia. (**D**) Due to the persistent radiologic–pathologic discordance driven by the BI-RADS 4C B-mode morphology, a segmental mastectomy was performed. The final surgical pathology specimen (H&E stain) revealed a significant B3-level upgrade, demonstrating a large radial sclerosing lesion (radial scar) that harbored SA and occult foci of atypical ductal epithelial hyperplasia (ADH). This case emphatically illustrates that highly suspicious B-mode imaging features mandate surgical excision to rule out adjacent high-risk lesions, regardless of a benign core biopsy and soft elastography findings.

Among the 21 patients subjected to surgical excision, 12 patients (57.1%) exhibited no pathological upgrade, confirming the initial diagnosis of SA or equivalent benign changes. However, 6 patients (28.6%) demonstrated a Level 1 (B3-level) upgrade, revealing occult high-risk lesions that fundamentally altered their long-term clinical management (Figure 1). Most critically, 3 patients (14.3%; 95% CI: 5.0–34.6%) demonstrated a Level 2 (malignant) upgrade. Cumulatively, the overall clinically significant upgrade rate within the surgical cohort was a notable 42.9% (9 of 21 cases; 95% CI: 24.5–63.5%). These wide Wilson score confidence intervals, a direct consequence of the modest surgical subgroup size, indicate that the true population upgrade rate could plausibly range from approximately one-quarter to nearly two-thirds of cases, and the point estimates should therefore be interpreted as hypothesis-generating rather than definitive.

**Figure 2:**
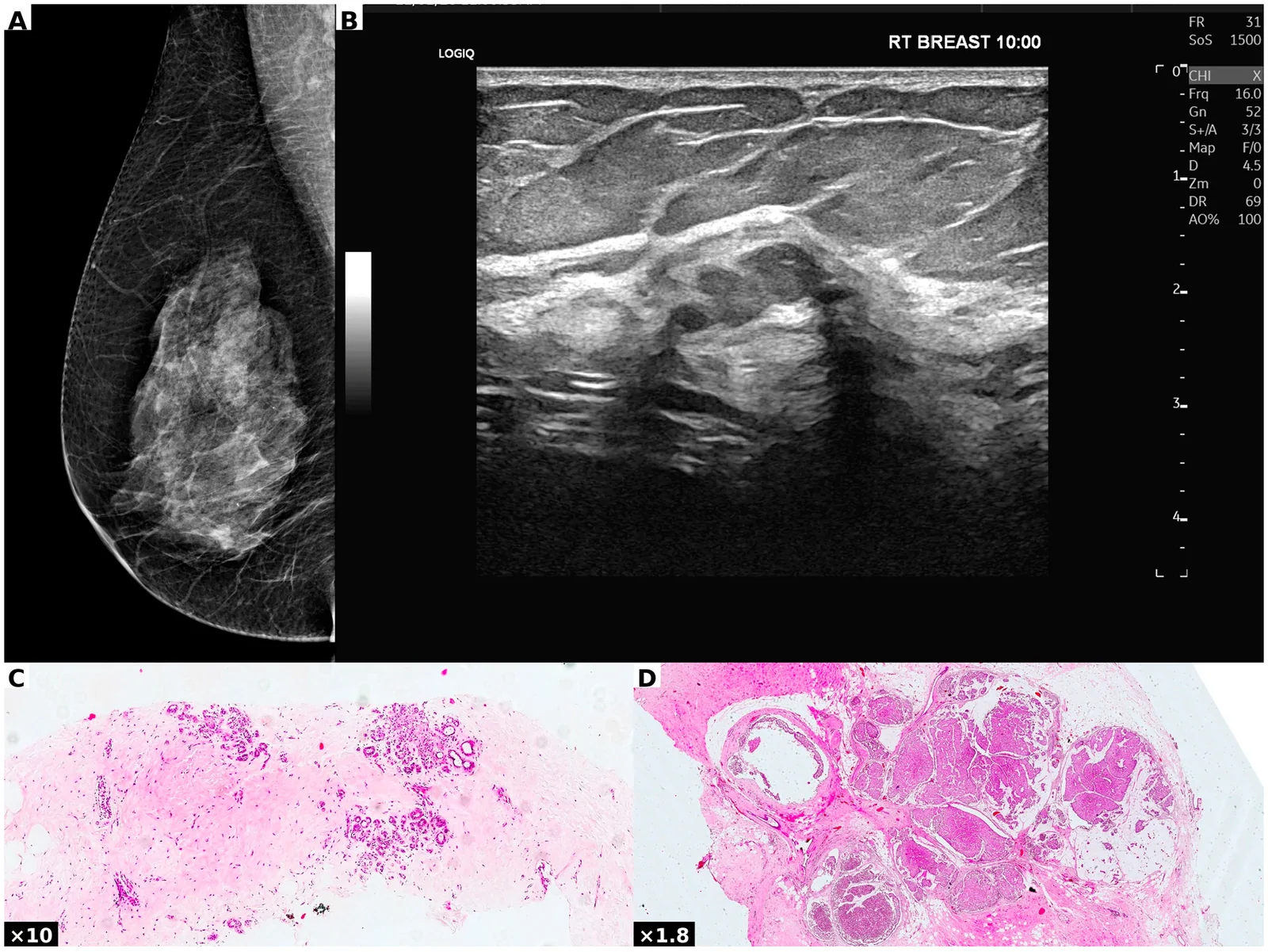
A 54-year-old female patient presenting with breast pain, demonstrating a malignant upgrade of a benign-mimicking lesion to a special histologic subtype. (**A**) Mammography (right MLO view) reveals an area of architectural distortion in the upper quadrant, which was also clearly demonstrated on digital breast tomosynthesis. (**B**) Targeted breast ultrasound shows an oval-shaped, lobulated, hypoechoic solid mass measuring 15 × 6 mm (BI-RADS 4) at the 10 o’clock position of the right breast, parallel to the skin. (**C**) Microscopic examination of the core needle biopsy specimen (H&E stain) yielded a benign diagnosis of sclerosing adenosis (SA) accompanied by microcalcifications and cystic changes, lacking atypia. Dual immunohistochemical staining for p63 and panCK confirmed preserved myoepithelial layers, which initially masked the underlying malignancy. (**D**) Due to the persistent architectural distortion and clinical-radiological suspicion, surgical excision was performed. The final surgical pathology revealed a 1.3 × 1.1 × 1.0 cm invasive mucinous carcinoma (pT1aNX, Bloom-Richardson Grade 2). The immunohistochemical profile of the tumor demonstrated strong luminal positivity with ER (100%) and PR (90%), Androgen receptor positivity (90%), HER2 negativity (score 1+), and a low Ki-67 proliferation index (10%). p63 staining confirmed the absence of a myoepithelial layer in the invasive areas. This case elegantly demonstrates that SA can mask special subtypes like mucinous carcinoma, which inherently exhibit lower-suspicion imaging features such as lobulated contours and benign-mimicking sonographic appearances.

**Figure 3:**
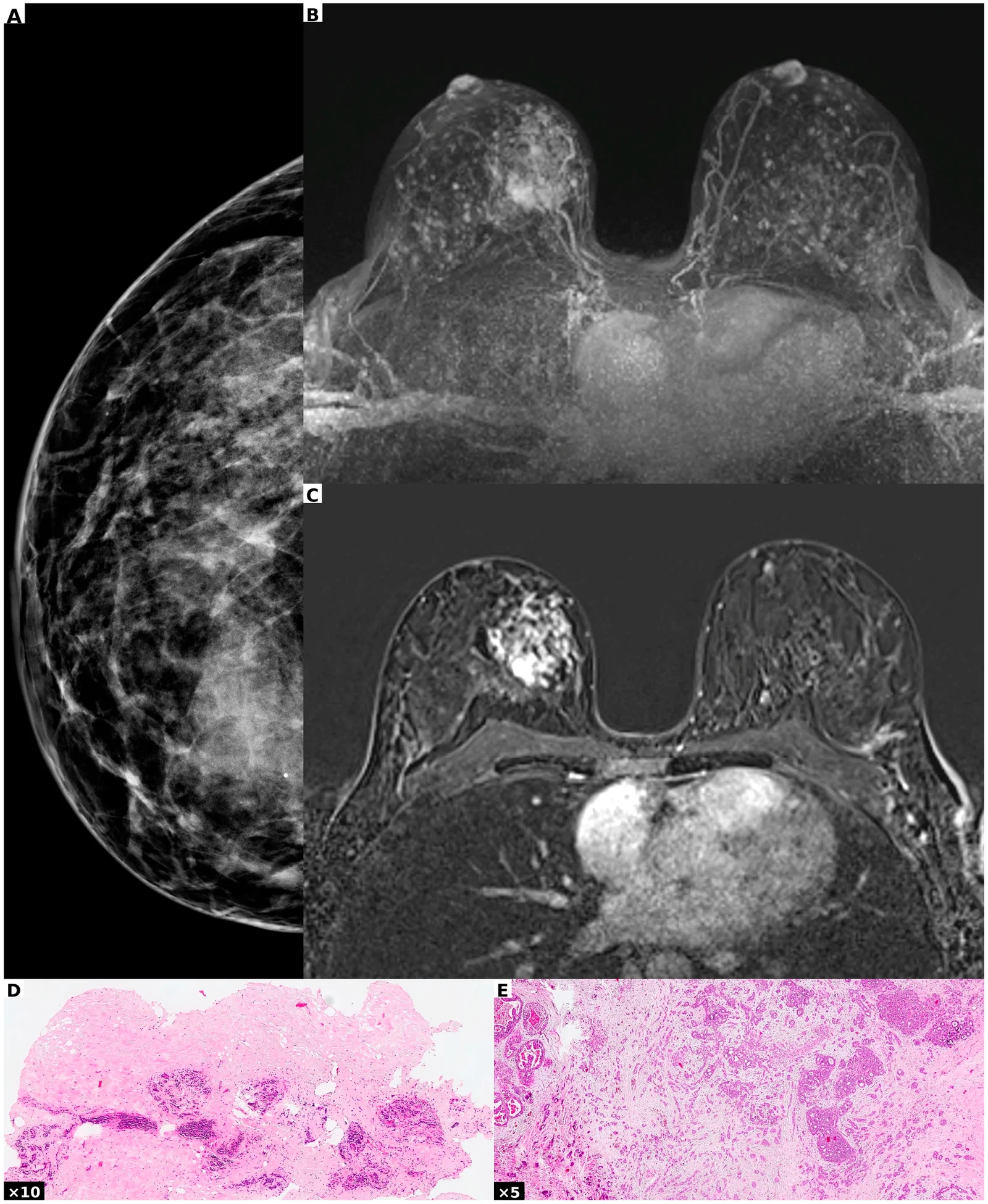
A 43-year-old female patient presenting with a 3-month history of a painless palpable mass, demonstrating a malignant upgrade due to sampling error. (**A**) Right breast mammography (craniocaudal view) demonstrates a focal asymmetric density with a few scattered microcalcifications. Correlative targeted ultrasound revealed only multiple dense complicated cysts without a suspicious solid correlate. (**B**,**C**) Dynamic contrast-enhanced breast MRI (MIP and axial T1-weighted post-contrast sequences) highlights a prominent area of asymmetric non-mass enhancement (NME) in the right breast, raising high clinical suspicion (BI-RADS 4B). (**D**) Microscopic examination of the MRI-guided vacuum-assisted biopsy (VAB) specimen (H&E stain) demonstrated fibrohyalinized breast parenchyma containing foci of isolated sclerosing adenosis (SA), without evidence of atypia. (**E**) Due to the profound radiologic–pathologic discordance, surgical excision was mandated. The final surgical pathology revealed an occult 6 mm (pT1b) histologic Grade 1 invasive ductal carcinoma (IDC) associated with a Grade II ductal carcinoma in situ (DCIS) component and corresponding microcalcifications. Immunohistochemistry demonstrated a Luminal A-like profile (diffuse E-cadherin positivity, ER 95%, PR 100%, HER2 negative, and a Ki-67 proliferation index of 12%). Notably, the background breast tissue exhibited a highly complex benign architecture, including widespread sclerosing adenosis, usual ductal hyperplasia, intraductal papilloma, cystic papillary apocrine metaplasia, and columnar cell change. This underscores how an intensely proliferative benign background can act as a desmoplastic shield, masking an adjacent low-grade carcinoma during core sampling.

Detailed analysis of the three malignant upgrades provided crucial clinical insights. Strikingly, two of the three malignant upgrades (66.7%) evolved from lesions that were explicitly classified as isolated SA without any cellular atypia on the initial percutaneous biopsy. These included the following:

1. A 54-year-old female presenting with BI-RADS 4A microcalcifications spanning 15 mm, whose final pathology revealed invasive mucinous carcinoma (Figure 2).
2. A 43-year-old female presenting with a 27 mm BI-RADS 4B solid mass, upgraded to invasive ductal carcinoma at excision (Figure 3).

**Figure 4:**
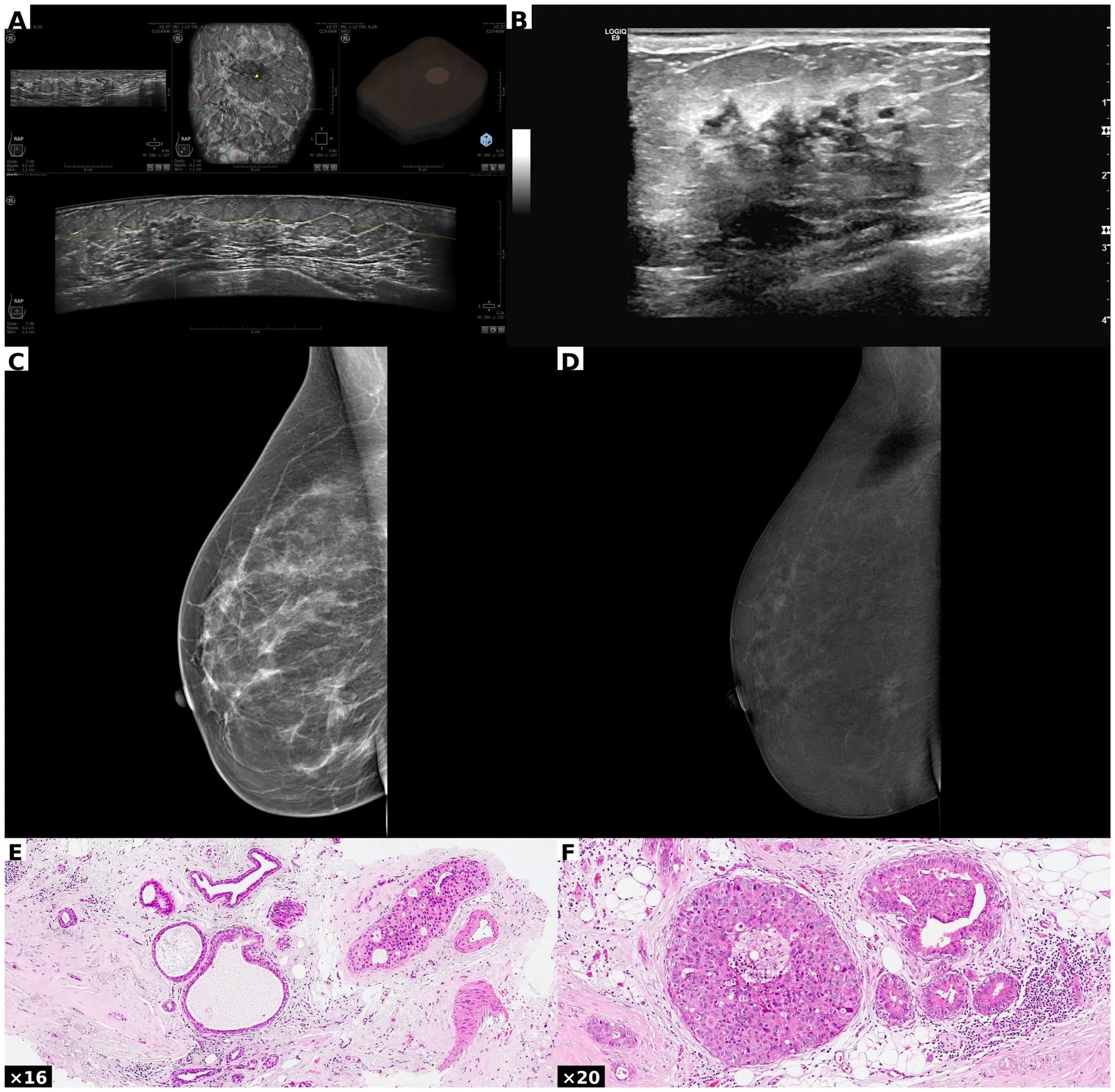
A 43-year-old female patient presenting with a 2-week history of focal breast pain, demonstrating the necessity of excision when atypia accompanies SA on core biopsy. (**A**,**B**) Automated Breast Ultrasound (ABUS) coronal reconstruction and targeted conventional ultrasound reveal an irregular, hypoechoic mass with internal microcystic spaces and pronounced architectural distortion. (**C**,**D**) Full-field digital Contrast-Enhanced Mammography (CEM) was performed (1.5 mL/kg iodinated contrast). The low-energy MLO image shows heterogeneous dense breast parenchyma (ACR Type C). The recombined iodine image (R-MLO#) highlights a highly suspicious 24 × 14 mm spiculated mass with irregular contrast enhancement and architectural distortion in the lower-outer quadrant of the right breast (BI-RADS 4C). (**E**) Microscopic examination of the core needle biopsy (H&E stain) revealed sclerosing adenosis (SA) with microcalcifications and a focus of atypical ductal hyperplasia (ADH) in a background of cystic apocrine metaplasia. Immunohistochemistry (p63+ and CK5/6+) confirmed an intact myoepithelial layer, validating the benign background. (**F**) The final segmental mastectomy specimen (H&E stain) demonstrated a malignant upgrade to solid-type ductal carcinoma in situ (DCIS, Grade II) arising in scattered foci within a 1.3 × 1.2 cm radial sclerosing lesion. Strong, diffuse nuclear positivity for ER and PR was noted in the DCIS. This case highlights that atypia identified within a complex multimodality imaging background mandates complete surgical excision.

The third malignant upgrade occurred in a patient with atypical SA (concurrent ADH) who was found to harbor Grade 2 DCIS upon complete excision (Figure 4). All three identified malignancies were estrogen- and progesterone-receptor-positive.

In stark contrast, all 13 patients managed conservatively with imaging surveillance remained radiologically stable over a median follow-up period of 16 months (range: 7–84 months, including three patients followed for only 7 months), with no delayed malignancies detected within the available follow-up window.

### 3.5. Predictive Factors for Upgrade

Of the 22 patients in the overall cohort whose CNB diagnosis was isolated SA, 12 subsequently proceeded to surgical excision and 10 were allocated to radiological surveillance; the univariate analysis that follows is therefore restricted to the 21-patient surgical subgroup, within which isolated SA on CNB was the CNB classification in 12 of the 21 excised cases. Univariate statistical analysis within this surgical cohort (*n* = 21) sought to identify clinical or imaging variables predictive of any upgrade (Table 1). Due to the constrained sample size of the surgical subset, no analyzed variable reached statistical significance.

† The surgical subgroup analyzed here (*n* = 21) is a subset of the full 34-patient cohort; see main text for the isolated-SA breakdown.

While not reaching strict statistical significance (*p* = 0.144), the median lesion size was notably larger in the upgraded cohort (22 mm) compared to the non-upgraded cohort (16.5 mm). Counterintuitively, isolated SA on CNB was also more prevalent among patients with an upgrade than among those without (7/9, 77.8% vs. 5/12, 41.7%; Table 1)—equivalently, of the 12 surgical patients whose CNB diagnosis was isolated SA, 7 (58.3%) upgraded, compared with 2 of the 9 patients with complex/atypical SA (7/12 vs. 2/9, *p* = 0.184); however, given the minuscule representation of atypical cases (*n* = 2) in this study, this finding must be interpreted as an anomaly of limited statistical power rather than a true biological divergence.

## 4. Discussion

Sclerosing Adenosis continues to present a profound diagnostic conundrum in contemporary breast radiology. Because SA involves the disordered proliferation of acinar, epithelial, and myoepithelial elements enveloped in a dense fibrotic stroma, its morphological footprint on ultrasound, mammography, and MRI is frequently indistinguishable from highly aggressive scirrhous carcinomas [12,13]. The primary objective of this investigation was to delineate the true upgrade rates of CNB-diagnosed SA within a symptomatic cohort and to scrutinize whether surgical excision remains an absolute necessity.

In this single-center, symptomatic cohort, the overall upgrade rate at surgical excision was 42.9% (95% CI: 24.5–63.5%), punctuated by a 14.3% (95% CI: 5.0–34.6%) upgrade explicitly to invasive malignancy or DCIS. These figures are substantially higher than the 1–2% malignant upgrade rates conventionally cited in the literature for isolated SA [3,4,14]. This marked discrepancy is clinically informative and driven by several critical factors.

Primarily, the phenomenon of verification bias (or work-up bias) is an inherent facet of this study’s retrospective design. The clinical decision to pursue surgical excision over surveillance was not randomized; it was heavily influenced by the degree of multimodality imaging suspicion, lesion size, and the multidisciplinary tumor board’s perception of radiological–pathological discordance. Consequently, the surgical subset (*n* = 21) was inherently enriched for higher-risk lesions from the outset. The 42.9% upgrade rate must therefore be contextualized as the risk profile of SA lesions pre-selected for surgery due to clinical suspicion, rather than the baseline risk of all CNB-diagnosed SA.

Secondly, and perhaps most crucially, our cohort was entirely symptomatic. The majority of the historical SA literature is derived from Western screening programs, which predominantly detect subclinical, asymptomatic microcalcifications [1,2]. Symptomatic presentation (palpable masses and focal pain) implies a distinct, often more advanced, underlying pathophysiology. The high rate of architectural distortion and solid mass formation observed in our cohort underscores that symptomatic SA frequently behaves as a dominant, space-occupying lesion, which inherently carries a higher baseline probability of harboring adjacent, unsampled malignancy.

The most notable finding of this study is that two of the three malignant upgrades originated from biopsies that were strictly classified as “isolated SA,” completely devoid of cellular atypia. This highlights the fundamental limitation of percutaneous biopsy techniques (including larger-volume VAB): sampling error. Sclerosing adenosis is frequently a macroscopic, heterogeneous “neighborhood” lesion that can act as a desmoplastic shield, effectively concealing adjacent foci of DCIS or small invasive carcinomas [15,16]. Critically, both of these “isolated SA” cases harbored imaging features that, in retrospect, constituted clear radiological–pathological discordance from the outset: one presented as a 27 mm BI-RADS 4B solid mass with mass-type MRI enhancement, and the other as BI-RADS 4A microcalcifications on mammography in a 54-year-old patient. In neither case was the imaging phenotype truly reassuring enough to accept a benign CNB result as concordant. When an imaging finding is highly suspicious (e.g., a 27 mm solid mass with architectural distortion) and the biopsy returns merely isolated SA, the radiologist must interpret this not as a definitive benign exoneration, but as a severe radiological–pathological discordance. This precise dynamic is why relying strictly on the absence of atypia in the CNB specimen is insufficient to preclude surgery when the imaging phenotype dictates otherwise.

To directly compare the imaging and biopsy characteristics of patients who progressed to malignancy against those who remained stable, we contrasted the three malignant-upgrade cases with the twelve surgical patients who showed no upgrade. The non-upgraded group was characterized by a smaller median lesion size (16.5 mm), a predominance of BI-RADS 4A assessments, and closer radiological–pathological concordance, such as vague sonographic shadowing that matched the dense stromal fibrosis seen at histology. By contrast, each of the three malignant upgrades displayed at least one element of imaging–pathology discordance despite a benign CNB result: a higher-suspicion BI-RADS 4B solid mass with mass-type MRI enhancement in one patient, BI-RADS 4A microcalcifications on mammography in another, and non-mass enhancement on MRI unmatched by a solid sonographic correlate in a third. No single imaging feature was uniformly present across the three malignant cases; rather, the recurring pattern was a mismatch between the degree of imaging suspicion and an ostensibly reassuring benign CNB result, reinforcing the value of a multidisciplinary, concordance-based triage strategy over reliance on histology alone.

The integration of advanced imaging modalities plays a pivotal role in these discordant scenarios. While breast MRI is unparalleled in its sensitivity, SA is notorious for producing false-positive enhancement, frequently exhibiting Type II (plateau) or Type III (washout) kinetic curves that closely mimic malignancy [8,9]. Consistent with this, among the 17 patients who underwent DCE-MRI, mass-type enhancement was the predominant pattern (*n* = 11, 64.7%). Regarding kinetic curves, Type I (persistent) curves were most frequent (*n* = 10, 58.8%), while Type II (plateau) or Type III (washout) curves—considered more suspicious for malignancy—were observed in 7 patients (41.2%), underscoring the substantial overlap between SA and malignancy on MRI. These MRI findings are descriptive rather than inferential: DCE-MRI was performed in only 17 of the 34 patients (a non-randomly selected subset), and kinetic curve pattern was not formally tested as a predictor of upgrade in our univariate analysis (Table 1). They should therefore be read as illustrative of the known MRI-SA overlap rather than as evidence that MRI systematically predicts upgrade in this cohort. Independent of formal predictive testing, this qualitative overlap supports our broader, case-based argument that aggressive MRI kinetics in the context of an isolated SA diagnosis on CNB are better interpreted as discordant than as reassuring. If a lesion exhibits aggressive MRI kinetics coupled with sonographic shadowing or mammographic architectural distortion, a CNB diagnosis of SA cannot be accepted as concordant. On this basis, we suggest that multimodality imaging suspicion be weighed carefully against a benign, isolated core pathology result when deciding between complete surgical excision or large-volume Vacuum-Assisted Excision (VAE) [17] and continued surveillance in such discordant scenarios, noting that this suggestion is based on a small, non-randomized surgical subgroup with wide confidence intervals around the observed upgrade rates.

Conversely, our data also lend provisional support to conservative management for appropriately selected patients. All 13 patients managed with imaging surveillance remained stable over a median follow-up of 16 months. These cases were predominantly BI-RADS 4A lesions characterized by imaging–pathology concordance (e.g., vague sonographic shadowing correlating perfectly with the dense stromal fibrosis of SA). This conclusion must, however, be tempered by the heterogeneity of the surveillance interval (range: 7–84 months), including three patients followed for only 7 months; such a short interval is insufficient to definitively exclude delayed malignant transformation, and the apparent stability of the surveillance arm should therefore not be read as proof of long-term safety but rather as an encouraging, hypothesis-generating observation pending longer, more uniform follow-up. For purely concordant, low-suspicion lesions, surgical excision inflicts unnecessary physical morbidity, psychological distress, and financial burden, reinforcing that surgical management should not be a universal mandate.

## 5. Limitations

This study is subject to several methodological limitations. The retrospective, single-center design introduces inherent verification and selection biases, as the triaging of patients to surgery versus surveillance was dictated by clinical judgment rather than a randomized protocol. The sample size (*n* = 34) is modest, which severely restricted the statistical power to conduct robust multivariable logistic regression analyses; thus, all derived *p*-values should be viewed as exploratory and hypothesis-generating. It should be noted that this cohort reflects an exhaustive query of a dedicated, high-volume breast radiology database over a six-year period (2020–2026). Sclerosing adenosis is frequently reported as an incidental, secondary finding accompanying a separate primary lesion (e.g., a carcinoma, fibroadenoma, or papilloma) that itself constitutes the indication for biopsy; such cases were not eligible for the present analysis. Because our inclusion criteria required SA itself—whether isolated or accompanied by other B3-level findings—to be the primary CNB diagnosis driving the biopsy, rather than an incidental accompanying finding to a separately diagnosed lesion, the eligible pool was inherently more restricted than the total burden of SA encountered in routine pathology reporting. We believe that the modest final sample size reflects this deliberately narrow, clinically meaningful case definition rather than incomplete case ascertainment at our institution. Additionally, the utilization of advanced imaging was heterogeneous (MRI and mammography were not performed on all patients), precluding a uniform, comparative multimodality analysis. The original CNB slides were not subjected to a formalized, outcome-blinded second pathology re-review independent of the surgical result; all initial “isolated SA without atypia” calls were rendered by subspecialty-trained breast pathologists as part of routine clinical care, but the absence of a structured, blinded re-adjudication is a further potential source of misclassification that should be addressed through prospective, blinded pathology review in future work. Similarly, formal inter-reader agreement for BI-RADS categorization among the interpreting radiologists was not systematically assessed, and residual inter-observer variability in BI-RADS assignment cannot be excluded. A related, unresolved limitation concerns the case-mix finding that isolated SA upgraded more frequently than complex/atypical SA (Table 1, Section 3.5); we have attributed this to limited statistical power given the small number of atypical cases, but an alternative, competing explanation cannot be excluded—namely, that some CNB calls of “isolated SA” partly reflect sampling misses of an adjacent, imaging-discordant lesion rather than a true absence of higher-risk pathology. Because this hypothesis was not formally tested (e.g., through blinded re-review correlating imaging discordance with CNB classification), it should be regarded as an untested competing hypothesis rather than a secondary observation and warrants dedicated evaluation in future, adequately powered studies. Furthermore, the follow-up duration within the surveillance cohort was markedly heterogeneous (range: 7–84 months); three patients had a follow-up of only 7 months, which is insufficient to exclude delayed malignant transformation and represents a meaningful caveat when interpreting the stability of the non-operative group. Finally, specific data regarding the precise needle gauge utilized for every procedure was not universally available, preventing an analysis of how larger-gauge vacuum-assisted biopsies might mitigate the observed upgrade rates [18,19].

## 6. Conclusions

In this symptomatic, single-center cohort, SA-spectrum lesions selected for surgical excision following a CNB diagnosis exhibited a substantially higher upgrade rate (42.9% overall, 14.3% malignant) than is traditionally reported in asymptomatic screening populations. Because this rate is derived exclusively from patients already triaged to surgery on the basis of clinical and imaging suspicion, it should be interpreted as the upgrade risk of this enriched, imaging-discordant subgroup rather than as the baseline upgrade risk of all CNB-diagnosed SA. The documentation of malignant upgrades arising from presumed isolated, non-atypical SA highlights the persistent threat of CNB sampling error and the critical importance of evaluating the entire lesion microenvironment. These findings argue against extending a blanket policy of non-operative management to symptomatic, imaging-discordant SA presentations resembling those in our enriched surgical subgroup, rather than to all CNB-diagnosed SA irrespective of imaging findings. Instead, clinical management must be rigorously individualized through a multidisciplinary framework that heavily weighs multimodality imaging–pathology concordance, precise BI-RADS categorization, and lesion size. Larger, prospective, multicenter trials utilizing standardized imaging protocols and minimally invasive complete excision techniques, such as vacuum-assisted excision (VAE), are warranted to definitively establish which SA cohorts can be safely relegated to imaging surveillance.

## Figures and Tables

**Table 1:** Univariate analysis of factors associated with any upgrade in the surgical cohort (*n* = 21).

Variable	No Upgrade (*n* = 12)	Upgrade (*n* = 9)	*p*-Value
Age, years (median)	41	43	0.432 *
Lesion size, mm (median)	16.5	22	0.144 *
Core samples, *n* (median)	6	8	0.105 *
Isolated SA on CNB, *n* (%)	5 (41.7)	7 (77.8)	0.184 **
BI-RADS 4C/5, *n* (%)	3 (25.0)	4 (44.4)	0.397 **
Any accompanying lesion, *n* (%)	7 (58.3)	2 (22.2)	0.184 **
Sonographic shadowing, *n* (%)	2 (16.7)	3 (33.3)	0.611 **

* Mann–Whitney U test. ** Fisher’s exact test.

## Data Availability

The data presented in this study are available on request from the corresponding author due to patient privacy and confidentiality agreements.

## Abbreviations

ABUS	Automated Breast Ultrasound
ADH	Atypical Ductal Hyperplasia
ALH	Atypical Lobular Hyperplasia
BI-RADS	Breast Imaging Reporting and Data System
CEM	Contrast-Enhanced Mammography
CI	Confidence Interval
CNB	Core Needle Biopsy
DBT	Digital Breast Tomosynthesis
DCIS	Ductal Carcinoma in Situ
DCE-MRI	Dynamic Contrast-Enhanced Magnetic Resonance Imaging
ER	Estrogen Receptor
HER2	Human Epidermal Growth Factor Receptor 2
H&E	Hematoxylin and Eosin
IDC	Invasive Ductal Carcinoma
IHC	Immunohistochemistry
Ki-67	Marker of Proliferation Ki-67
MG	Mammography
MLO	Mediolateral Oblique
MRI	Magnetic Resonance Imaging
NME	Non-Mass Enhancement
PR	Progesterone Receptor
SA	Sclerosing Adenosis
SMMHC	Smooth Muscle Myosin Heavy Chain
TDLU	Terminal Duct-Lobular Unit
US	Ultrasound
VAB	Vacuum-Assisted Biopsy
VAE	Vacuum-Assisted Excision
